# “Pouring their heart out in Sainsbury’s”: qualitative study of young people’s, parents’ and mental health practitioners’ experiences of adapting to remote online mental health appointments during COVID-19

**DOI:** 10.1186/s12888-023-05126-8

**Published:** 2023-09-02

**Authors:** Lucy Biddle, Jane Derges, Bethany Cliffe, Rachael Gooberman-Hill, Myles-Jay Linton, Paul Moran, Helen Bould

**Affiliations:** 1 Population Health Sciences, Bristol University Medical School, Canynge Hall, 39 Whatley Road, Bristol, UK; 2 The National Institute for Health Research Applied Research Collaboration West (NIHR ARC West) at University Hospitals Bristol and Weston NHS Foundation Trust, Bristol, UK; 3 Centre for Academic Mental Health, Bristol University Medical School, Oakfield House, Oakfield Road, Bristol, UK; 4 Biomedical Research Centre at University Hospitals Bristol and Weston NHS Foundation Trust, Bristol, UK; 5 Translational Health Sciences, Bristol University Medical School, Learning and Research Building, Southmead Hospital, Bristol, UK; 6 Elizabeth Blackwell Institute, University of Bristol, Royal Fort House, Bristol, UK; 7 Gloucestershire Health and Care NHS Foundation Trust, Gloucester, UK; 8 Medical Research Council Integrative Epidemiology Unit, University of Bristol, Bristol, UK

**Keywords:** Adolescents, Young adults, Telemental health, Telepsychiatry, Qualitative, COVID-19, Mental health services

## Abstract

**Background:**

During the COVID-19 pandemic, technologies such as videoconferencing were used to deliver mental health appointments remotely online. For many people, this was a change from previous methods of mental healthcare receipt and delivery. We aimed to explore in-depth how practitioners, young people and parents in the UK experienced this transition.

**Methods:**

We used qualitative methods to collect data, triangulating between free-text online survey data (*n* = 38), focus groups (*n* = 5) (3 young adult groups (total *n* = 11); 2 practitioner groups (total *n* = 7)), and semi-structured interviews (practitioners *n* = 8; parents *n* = 4). Data were analysed using reflexive thematic analysis.

**Results:**

Participants held mixed views about remote appointments, which were encompassed within the five themes of: home as clinic; disrupted therapeutic relationships; difficulties with engagement; uncontained risk; and scope of care provision. While appointments at home could be regarded as more comfortable, naturalistic and accessible, it was also recognised that remoteness compromised practitioner control with consequences for their ability to monitor patient engagement, manage risk and ensure confidentiality when others were present in the home. This could create an additional burden for parents as they tried to facilitate appointments but felt unsupported in this role. Relatedly, remoteness was seen to hinder interpersonal communication, formation of trust, communication of empathy and opportunities to observe body language, all of which were deemed important to building and maintaining effective therapeutic relationships. Despite this, others thought the anonymity of a remote exchange may allow earlier disclosure. There was disagreement as to whether remote provision narrowed or expanded the scope of practice.

**Conclusions:**

While some had positive views of remote mental health appointments, others found them challenging. Findings highlight key areas requiring attention and mitigation in future offerings of remote provision, namely: risk management, parental burden, and problematic engagement.

## Background

 During the COVID-19 pandemic, many mental healthcare practitioners and patients had to rapidly adapt to remote services made accessible via technology such as telephone or videoconference platforms [1, 2]. Before the pandemic, the UK’s National Health Service (NHS) had outlined strategies for digitising care to increase service capacity and meet increasing demand [3]. However, uptake was relatively low among both clinicians [4, 5] and young people [6]. Evidence from systematic reviews indicated the effectiveness of telephone-delivered therapy for depression and anxiety [7, 8], however a range of barriers to implementation were reported; notably gaps in practitioner knowledge and skills, a lack of standardised guidelines [5], and negative perceptions and resistance to this mode of service delivery from both practitioners and patients [5, 9]. A need for behaviour change was highlighted at a practitioner, patient and service level [5].

Similarly, while identified as a feasible way forward for young people’s mental health [10], review studies noted limitations in the evidence-base exploring the effectiveness and acceptability of telemedicine for this age group [11]. Particular concerns surrounding use of telemental health with young people have focused on the complexity of establishing and sustaining meaningful therapeutic relationships [10, 12], ensuring access to private space and protecting confidentiality [10, 13], and concerns about ensuring safety and adequate risk assessment [10]. Given this context, and with a view to considering future service provision, it is fruitful to explore stakeholders’ experiences of the adoption of remote mental health support during COVID-19 where the transition to telehealth occurred abruptly, without guidance and not limited to specific interventions, but for a full range of service provision in place of usual appointments.

Research exploring remote mental health provision (and more general remote healthcare provision [14]) during COVID-19 has tended to use primarily survey methods, to focus on delivery to adults, and on the experiences of practitioners/ specific practitioner groups such as family therapists or psychotherapists (e.g [15–21].). While many practitioners in these studies report positive experiences, which they attribute to having greater flexibility, being able to continue providing support, and patients feeling more able to share in sessions; concerns are also raised about difficulties assessing patient safety, weaker therapeutic relationships, impact on non-verbal communication, therapist fatigue, and possible digital exclusion [14–16, 18, 19]_._ Available qualitative data also highlight difficulties—namely attending to nuanced expressions of emotion, working with groups as opposed to individuals and ensuring a confidential exchange [15, 21, 22]—while also providing evidence that remote appointments can be perceived as acceptable by both clinicians and patients [17].

Research on patients’ experiences of receiving online mental health treatment during covid is minimal [23] and few studies have explored young people’s perspectives on receiving support in this way, despite some evidence that in the UK the shift to remote appointments during COVID-19 was more pronounced for children and adolescents than other age groups [2]. An Australian survey of 308 young people and 92 clinicians found young people described the transition to telehealth as more beneficial than clinicians and expressed some interest in continuing to use this post pandemic [20]. While encouraging, these results afford little insight into the lived experience of young people during this time and qualitative research with adult service users has described their experiences of remote care as only ‘adequate’ or ‘tolerable’ due to difficulties forming a bond with their clinician and feeling immersed in therapy at home [22].

Less still is known about the experiences of parents. During the pandemic many parents experienced distress and burnout associated with home schooling and homeworking alongside limited opportunities for leisure [24–27]. Adjusting to remote appointments for children with mental health support needs may have added complexity or made attending appointments more accessible.

This study aimed to understand how young people, parents and mental health practitioners in the UK experienced receiving/ providing remote mental healthcare during COVID-19 restrictions. In this study, young people encompassed children, adolescents and young adults aged up to 24 years and we explored any remote delivery via online videoconferencing and telephone in place of a usual face-to-face offering. This included Child and Adolescent Mental Health Services (CAMHS) and student well-being services and a full range of appointment types and therapies (individual, group and family). Young people in the UK with mental health difficulties can be supported within the National Health Service (NHS) by Child and Adolescent Mental Health Services (CAMHS), usually until 18 years, though a transition to adult services may commence between the ages of 16 and 24 years. CAMHS, like other services experienced an abrupt transition to remote support with the onset of COVID-19 lockdown in March 2020.

## Methods

Qualitative data were collected from young people, parents of young people receiving mental health care, and mental health practitioners as part of a broader mixed-methods project exploring issues relating to digital technology and mental health in young people [28, 29]_._ Various data sources were synthesised: focus group discussions, semi-structured interviews and open-text questionnaire data (Table 1).

**Table 1:** Summary of data sources

Data source	Population	Method of data collection	Format of data obtained	Identifier in presentation of results
1	Young people aged 14–24 years living in UK	Online survey	Open-text questionnaire responses	YPs
2	Young people aged 16–24 years in the UK	Focus groups	Verbatim transcripts	YPfg
3	UK Parents supporting young person under 25 years with mental health difficulties	In-depth one-to-one interviews	Verbatim transcripts	P
4	Mental health practitioners, UK-based, supporting young people under 25, all sectors	In-depth one-to-one interviews	Verbatim transcripts	MHPi
5	Mental health practitioners, UK-based, supporting young people under 25, all sectors	Focus groups	Verbatim transcripts	MHPfg

### Recruitment and data collection

#### Young people

Data from young people were collected using an online survey and focus group discussions.

The survey was administered between June and December 2020 [28]. All young people aged 14–24 years and resident in the UK with online access were eligible. Participants were self-selecting, recruited via advertisements placed on social media, the young person’s mental health app ‘Tellmi’ (https://www.tellmi.help), and through networks within third sectors organisations (including youth groups), local schools, and universities. The survey incorporated questions on digital technology use during COVID-19, and where applicable participants were asked to provide a open-text answer to the question: ‘if you have received online therapy in place of usual face-to-face services due to COVID, please describe your experiences (including likes and dislikes)’.

Focus groups were run with young people in the UK who were English-speaking, aged between 16 and 24 years and self-reported mental health difficulties for which they had sought help from services (statutory, private or charity sector) [29]. Those currently receiving inpatient care were not eligible due to possible risks associated with their participation. Potential participants were identified using the strategies described above for the survey and by inviting eligible survey participants who had expressed interest in follow-up research. Interested individuals completed a brief online screen. All eligible participants were invited.

#### Parents

Parent data were collected using one-to-one in-depth interviews. They were invited to take part via targeted advertisements placed on Twitter and emailed to groups providing a forum for parents of children with mental health difficulties. Parents were eligible if they lived in the UK, were English speaking and were supporting a young person (under 25 years) receiving remote appointments during the pandemic. Focus groups were not conducted with this sub-sample because recruitment was sporadic.

#### Practitioners

One-to-one interviews or focus groups were conducted with UK-based mental health practitioners who currently delivered mental health care to children or young people aged up to 24 years [29]. Qualified clinicians from statutory National Health Services (NHS), student well-being services, third sector mental health organisations and the private sector were eligible. Participants were self-selecting and recruited via advertisements disseminated on social media, among professional networks available to the research team, to participants of a prior study survey [28] who had expressed interest in further involvement, and through snowball sampling. Interested individuals were screened and all those who were eligible were invited. A choice of focus group or one-to-one interview was offered to accommodate clinical working schedules.

Qualitative data collection took place online using secure video conferencing software. Focus groups were facilitated by LB and JD and lasted approximately one hour. Interviews were conducted by JD. Practitioner interviews lasted approximately 45-min and parent interviews between 60 and 90 min. Young people and practitioner groups/ interviews explored a range of issues relating to young people’s digital technology use and mental health [29], while parent interviews focused exclusively on experiences of remote mental health care provision for their child. Participants were encouraged to talk freely and to relay their experiences in their own terms, alongside use of a brief checklist of areas for probing. Data were collected in late 2020.

### Data analysis

Focus groups and interviews were audio-recorded and transcribed verbatim. In the case of young people and practitioner interviews, data specifically relating to remote appointments was extracted to new files for analysis. Transcripts of parent interviews were worked upon in their entirety. Reflexive thematic analysis [30] was used as a flexible method for exploring participants’ lived-experience, views and perceptions, and identifying patterns of meaning across datasets. This involved following six analytical phases to generate and map codes, then conceptualise, review and define themes. Segments of text were labelled according to their content and meaning, using codes derived inductively from the data. Coding was carried out collaboratively between LB and JD, who both independently coded 20% of the data, then met to discuss interpretation and agree codes. JD subsequently coded remaining young people data and LB coded practitioner and parent data. Data from the three sample groups were coded simultaneously to promote common coding as far as possible. A list of codes was derived from across the data sets and following discussion about the content of each code, these were organised by LB and JD into higher-level conceptual themes. Descriptive accounts were prepared for each sample group to review and explore similarities and differences within each theme.

Open-text survey responses were analysed in a similar manner, using open-coding to label text according to the ideas expressed. Coding was again collaborative and undertaken by LB and JD. The codes generated aligned entirely with those identified in the focus groups, allowing the two datasets to be merged at the point of higher-level coding.

Quotations are presented with the notation shown in Table 1 (above).

## Results

There were 320 responses to the young person survey. Of the 48 participants (15%) who reported receiving online mental health care in place of usual face-to-face services during COVID-19, 38 (79% of those receiving online care) provided free-text responses (Table 2). These participants were mostly female (71%) and had a median age of 19 years (range 14 – 24 years). Characteristics of the total survey sample are reported elsewhere [28].

**Table 2:** Characteristics of survey participants providing free-text comments

Characteristic	N (%)
**Age (years)**	
Median (IQR)	19.0 (16.3—21.2)
< 16	7 (18.4)
16–17	10 (26.3)
18–25	21 (55.3)
**Ethnicity**	
White	33 (86.8)
Black/ African/ Caribbean/ Black British	1 (1.6)
Asian/ Asian British	0 (0)
Mixed/ Multiple ethnic groups	3 (7.9)
Other ethnic group	1 (2.6)
**Sexuality**	
Heterosexual	19 (50)
LGB +	18 (47.4)
Prefer not to say	1 (2.6)
**Gender**	
Female	27 (71.1)
Male	6 (15.8)
Any other (including non-binary)	5 (13.1)
**Current Occupation** ^**a**^	
Any education	29 (76.3)
Any employment	17 (44.7)
Not in education, employment or training (NEET)	5 (13.1)

^a^ Categories not mutually exclusive

Eleven young people expressed interest in participating in a focus group. All were eligible and took part, resulting in three groups composed of six, three and two participants. Seventeen practitioners responded to adverts but two were not eligible (trainee, unqualified). The remaining 15 were invited and took part, seven attending focus groups (*n* = 3 group 1, *n* = 4 group 2) and eight attending a one-to-one interview. The young people were females and one non-binary participant, aged between 16 and 24 years and reporting a range of mental health difficulties. Mental health practitioners were from varying settings and had been practicing for between 5 and 43 years (median, 18) (Table 3). Five parents expressed interest in participating; four took part. All were mothers. Together, they had supported five young people (3 male, 2 female), receiving remote appointments at the ages of 10, 16, 19, 20 and 23 years due to presenting issues of anxiety, emotional dysregulation, self-harm behaviour, eating disorder, depression and bipolar disorder.

**Table 3:** Young people and practitioner participant characteristics

	**Young people (** ***n*** ** = 11)**	**Practitioners (** ***n*** ** = 15)**
**Age** (years) median (range)	20 (16–24)	Not collected
**Gender, n (%)**		
Female	10 (91)	11 (73)
Male	0 (0)	4 (27)
Non-binary	1 (9)	0 (0)
**Ethnicity**		
White British	9 (82)	14 (93)
Black British	1 (9)	0 (0)
Asian British	1 (9)	1 (7)
**Self-reported diagnosis**		
Anxiety and depression/ depression	5 (45)	Not applicable
Suicidal thoughts/ self-harm	1 (9)	
Eating disorder	4 (36)	
Borderline Personality Disorder	1 (9)	
**Current clinical role**		
CYPMHS Psychiatrist	Not applicable	3 (20)
CYPMHS Psychiatry advanced training registrar		2 (13)
CYPMHS Specialist physician		1 (7)
CYPMHS Psychologist		1 (7)
CYPMHS Family therapist		1 (7)
CYPMHS Nurse		2 (14)
Drugs and Alcohol worker		1 (7)
Student wellbeing services		2 (14)
Third sector practitioner		2 (13)

*CYPMHS* Children and young people’s mental health services

### Thematic analysis

We conceptualised five key themes. Participants focused primarily on issues relating to i) appointments being situated within the private space of the home, this being the typical site of remote appointments (‘home as clinic’) and ii) on the impact of remoteness on therapeutic relationships and interpersonal communication between practitioner and patient (‘disrupted therapeutic relationships’). This prompted discussion about the quality and safety of a remote exchange centring around the further three themes of iii) ‘difficulties with engagement’, iv) ‘uncontained risk’, and v) the ‘scope of care provision’ where this is provided remotely.

#### Home as clinic

Young people and parents commonly contrasted the familiarity of their home environment to appointments held in ‘unknown’ formal settings. Home could afford feelings of comfort, relaxation and safety, which could facilitate participation. This included having ‘a hot drink’, drawing on comforting objects and having supporters nearby.

I deffo like [online support group] better – can play with playdoh/ kinetic sand at the same time. (YPs).

[Daughter] asked if it would be okay to do [appointment] in her own room... I feel that is working because she’s in her safe space and she’s able to engage one-to-one without feeling threatened because she’s in her comfort zone, she’s got her cushions around her and she’s very often got the cat because he is like her comfort cat (P3)

One parent described ‘moving’ therapy to capitalise on this opportunistically:

We’ve got like a little snug room downstairs and [son] sort of sees that as his space... he tends to be in it most of the time. And so he’d been watching something on TV and so I went and sat next to him with the laptop there (P1)

Online appointments also presented a welcome alternative for young people whose symptoms made it difficult for them to leave the house or meet others.

I have severe anxiety and depression so going out is such a big deal for me… for me it’s [remote appointments] a lot of pressure taken off. (YPfg1)

Some practitioners noted that connecting with a young person at home could create a more naturalistic exchange, affording insights into the living environment, unexpected inroads into private narratives or opportunities for rapport building.

I’ve met so many pets and stuff led to stories and actually told us information… it’s opened up a whole number of possibilities. (MHPfg2)

However, for some young people, bringing appointments into the home encroached upon a space and identity they preferred not to associate with their mental health difficulties, and two described how ‘meeting’ at home denied them the helpful “ritual of going to appointments” (YPs):

I’m not in the headspace, I’m just at home, which I don’t like. Part of that is just getting the nervous energy out by walking there… for the whole morning I was just not doing anything, fretting about [appointment]. But if I like had to get a shower and go there that would be better for me. (YPfg2)

All participants agreed that having a confidential space to join appointments at home could be problematic, especially if presenting problems involved family members or young people. Fear of being overheard or witnessed as a patient was a barrier to disclosure:

It was much less private due to being in the same building as parents, so I couldn’t talk about some things. It didn’t help at all (YPs)

Parents were required to manage space to enable appointments:

Her room’s next door to her sister’s. So, I explained to her sister that on a Wednesday between 10 and 12 you can’t go in your bedroom… Luckily the weather was quite nice so we could sit out on the balcony (P2)

Practitioners noted how holding appointments in a chaotic or non-private environment could result in a suboptimal exchange.

Many of the people I work with have big families. There are loads of kids running round… then you’re going to be careful about which kinds of things you ask… often people can’t talk as well. They’ll be like, ‘[vocalises muffled noises]’, and I can hear there’s loads going on for them (MHPi4).

#### Disrupted therapeutic relationships

The impact of remoteness on communication between patient and practitioner was a common concern. Parents and young people described struggling to ‘open up’ to *‘a stranger talking over a screen’* (P1), commenting *‘I feel like we don’t really know each other as well as if it was in person’* (YPfg3). Similarly, practitioners questioned whether it was possible to establish or maintain comparable relationships to those built offline:

Students that have been referred since lockdown, we’ve said as a team, we’re all struggling to remember them…there’s definitely a feeling that we’re not as close, as connected… It’s quite sad not seeing students that I was seeing face-to-face and going to ringing and video calling them and that does change the relationship that was already there (MHPi8)

You can build the relationship with people over a computer screen but whether it’s the same, I’ve got no idea. (MHPi6)

Some participants explicitly linked in-person contact with trust:

With a lot of therapists, it’s about that really deep trust relationship and maybe not being able to see a person face-to-face despite it being on Zoom, for stuff like that I find it very difficult (YPfg2).

I’m so concerned of the importance for me as well as for the young person of that relationship on a face-to-face level for engagement, for privacy, for connection and for trust. (MHPfg2).

Young people also discussed how remoteness may hinder practitioners from imparting other important components of a therapeutic relationship such as empathy, attention and reassurance.

It’s hard [online] because you don’t really understand if someone’s like showing you empathy, like you don’t have that. They can tell you what you want to hear but you don’t know if they really care because you’ve not got the eye contact, like face to face. I think that’s vital (YPfg1)

I prefer face to face just because I think it’s easier to read body language and so it’s more, like, reassuring (YPfg1)

However, a contrasting observation by one practitioner was that distance between practitioner and patient can also be advantageous:

Nonetheless I mean, yeah, like I said people are disclosing much sooner I feel than they would otherwise, so there’s something around the sense of anonymity (MHPi4).

Of fundamental concern was the potential for remoteness to disrupt non-verbal communication and a practitioner’s observation of their patient. Some practitioners expressed this as being denied part of their clinical toolkit, which removed the opportunity to attain a more nuanced understanding:

I’ve been a child psychiatrist for twenty years and I’ve spent all that time honing my skills in working with people face to face … in the room, working with the non-verbal communication, you know, the unsaid and all of that, that feeling that you get about don’t go there, all of that is in the room. Doing it online is completely new (MHPi6)

I get more out of a discussion being in a room with someone, thinking about what’s that emotion that I can feel, what’s being transferred from this young person, how can I use that. Silences don’t feel so awkward because you’ve got all that body language and the non-verbal communication, it just gets lost on a screen (MHPfg2).

Parents and young people similarly acknowledged these limitations in both new and established relationships:

You have eye contact over a screen, but it’s not the same as sitting in a room and I suppose [practitioner] would observe things about [son] like, ‘Oh J, you’re sitting on the edge of the sofa now, what’s wrong? (P1)

Soon as we were able to go face to face that worked better … being able to read body language and like signs and as your clinician gets to know you. I know with my therapist, she knows from certain things I’ll be doing with my hands for example, she can tell if I’m like anxious or whatever. So, if I’m sitting [on Zoom call] and she can’t see anything necessarily, it’s really difficult (YPfg2).

Other examples implied a good therapeutic relationship can be built remotely. Some suggested the personal characteristics of the practitioner may be of heightened importance while others alluded to a learning curve:

At the beginning, I definitely found it easier working online with the people that I knew already. But that’s just because I wasn’t very practiced at working online. But actually, some of the newer people I’ve only ever worked with them online and it’s been absolutely fine and they’re doing really well (MHPfg2)

#### Difficulties with engagement

Challenges with engagement were commonly reported, both in relationships that had commenced remotely and those that were pre-existing. A direct difficulty was where a young person refused to join an appointment or dropped out.

[Son] was sort of sticking his feet up in front of the camera and wriggling around the bed and trying to kind of hide behind me…Within about a minute of the counsellor asking him how he was, had he thought of a goal, he said, ‘I don’t want to bloody do this’, slammed the laptop shut and ran off (P1)

Parental engagement could also be difficult:

A lot of young people have found it really easy to not engage, either not answer their phone, or video calls, or text a few minutes before, say they're too busy… Parents as well! … they can say, 'no, not today. We're busy,' and not quite appreciate the importance of the support. (MHPfg1)

All participant groups noted that the nature of remote appointments permits avoidance – though some young people also recognised this was undesirable:

If you’re sitting in a room with someone, it’s very hard to walk away isn’t it. ‘Bye bye’. That’s the downside to [remote appointments]. (P2)

If I go face-to-face I’m less likely to back out than online because it’s easy to just disappear (YPfg2).

I did not like it as I could easily miss sessions without having to explain myself (YPs).

Poor engagement was explained in various ways—a virtual appointment: cannot hold the attention of a child who is *‘easily bored’* (P2); may cause anxiety; exacerbates pre-existing difficulties in relating to a practitioner; or is confronting because the young person has to ‘witness’ themselves as a patient, seeing their own expressions reflected on the screen. Some participants also described it resulting from the content of what was offered remotely, for example, usual therapy being replaced with an inappropriate online alternative, or the young person finding virtual therapy distressing but being unsupported due to the remote context. For one participant, this compromised future care:

Now she doesn’t do group because she can’t cope with it online. It’s quite easy on zoom if you’re not coping to shut that laptop down. Or she’d mute it and turn the camera off and just say I’m listening, but she’s not... and now she’s not allowed any therapy because she couldn’t cope with zoom. So now that’s it, finito. She won’t get a one-to-one. If it had been different, if she’d been going to a group there would have been more support. Virtual support didn’t cut it I suppose… the only contact she had was from the guy that runs it and he said if you don’t come next week, it counts as a fail… they kick you off (P2)

However, one practitioner offered the contrasting interpretation that their patient’s disengagement was a positive sign that the young person was acting to preserve the therapeutic relationship by disengaging where they found therapy unhelpful:

One client often hangs up on me when they feel that, ‘actually you know what? I’ve had enough of this conversation now and I’m taking control’. But every week we speak again. And I don’t know whether that would happen if they knew they had to sit through 50 minutes of me kind of asking them questions [laughter] (MHPi4)

Where engagement was problematic, parents could inadvertently become a conduit, engaging for the child or adopting a quasi-professional role in an attempt to assist therapy or plug gaps in services. This created tension and fears about inadequacy.

[Daughter] was dead nervous because obviously she’d never done video calling… So I said, ‘well I’ll sit with you’… she was really anxious the following week when she was going to do it by herself to the point where she wanted to throw up... It got to the point where I put an I-phone on loudspeaker and [she] would stand beside me but again she wouldn’t engage. She’d be like “yeah” “no” and she’d be looking to me all the time to answer for her…. Her caseworker actually said to me [daughter] is very fortunate because she has got you and we know she’s being fully supported and I thought well that puts a lot of pressure on me… you’re just thrown back to the lions basically. It was a very scary time. (P3)

Other problems with engagement were more subtle, exhibited by patient behaviours such as not creating sufficient time or space for appointments, having divided attention, behaving informally, opting for telephone rather than video calls, or attending sessions with cameras switched off.

They’ll go, ‘I'm at a supermarket’ and you know, they're walking round Sainsbury’s pouring their heart out to you. You go, ‘are you sure?’ ‘Yeah, yeah, fine, carry on’ … one of my colleagues had a video call and the [young person] was in bed, in her pyjamas which weren’t particularly covering and then it turned out that somebody else was in the room as well and he was a bit like, ‘look’ I’ll ring you back in 10 minutes, you know, you need to get dressed, to get up and maybe we can have a chat when it’s just you in your bedroom’ (MHPi8)

Some young people, who I hadn't met before, refused to put their camera on during a risk assessment which was extremely difficult. I think that is hugely problematic at the moment. (MHPfg1)

Such digressions from usual doctor-patient encounters could be difficult for practitioners to manage or interpret, particularly where there was no prior relationship, and could raise safeguarding concerns, discussed in theme iv, below):

There's very few [young people] that will sit, say like [other focus group participant] is sitting where you can feel you're having a conversation…. Say we're on a video call and they're on their phone or they've got their game station playing, I found that really tricky. Where the relationship is already there, it's easy to say, 'Pick it up and talk to me,' but with some, it's very, very hard because you don't know if they actually need that distraction before they can engage. (MHPfg1)

Often times people are eating or that kind of thing that because it’s, you know, you’re not face-to-face, it’s not so formal and you can kind of lounge around and snack. And it’s quite hard to manage those sorts of frames and boundaries and for it to feel professional.

#### Uncontained risk

Participants discussed how the practitioner’s physical absence afforded them less control. A primary concern was around the safety of provoking emotion yet being unable to intervene sufficiently to manage this.

Speaker 1: Our therapy is often going to raise their emotions [other participants expressing agreement]. Yeah, and then we press the off button.

Speaker 2: Normally if at the end of the session someone was stressed, someone in the nursing team would be able to help them through that. Some of them [receiving remote therapy], their parents are at work, would be on their own. (MHPfg2).

These concerns were felt acutely by parents who felt left to sweep up strong emotions.

[Daughter] said “Mum [online group] was really hard. Everyone was talking about things they really shouldn’t be”…It got to about midnight and she was still not coping ... On the second week she came downstairs and said “I’ve logged out, I can’t do it” … I said explain that they’re talking about things that are quite triggering ..,But they were very much like ‘well it’s group, you’ve got to do it’. (P2)

Two parents noted that remote practitioners are also less able to monitor changes of mood during an appointment, especially if cameras are turned off.

I’d literally be holding the phone and [daughter]’d be stood there in tears while [caseworker] had been talking …because [caseworker] couldn’t see her, she didn’t know those words she was saying were impacting so badly. If she’d been face-to-face, she’d have seen that the minute she said that, that’d triggered that emotion (P3)

While some young people also expressed these concerns, their comments were juxtaposed with alternative perspectives about the safety and comfort of home as a setting for therapy (above). The helpfulness of being surrounded by coping strategies was identified as a positive aspect of remote appointments.

I found online therapy a lot more helpful because I found [therapy] like quite a risky situation for me. Because I live with loads of things in my head it would be really hard to go home. So, I found it a lot easier and not only that I’ve got my room and I’ve got my blanket if I want to just go to bed and just lie in bed after. I can speak to my family, yeah so being in like my safe space is actually a lot more helpful. (YPfg2)

Practitioners extended discussion to the challenges of managing high-level risk and disclosures during remote sessions.

You have less control of what you're doing, so for example if someone has said they're about to kill themselves… people can handover their means of suicide, which I have done in the past. You can’t do that remotely (MHPi1).

Practitioners were at differing stages in anticipating and responding to the challenges raised:

I had a session a couple of weeks ago where a young person went to get a knife in session so again it was like that wouldn’t happen [in service setting]… without wanting to sound too dramatic, the worst case scenario was someone harming themselves on screen whilst being completely unable to stop it. So that’s one thing that’s stuck in my mind and we’re [the team] still thinking about it… (MHPfg2)

I have to be quite clever about using messages as well as visuals and stuff if I think there's something else going on [in home environment]... If it’s an acute risk, I kind of come up with something quite crazy, something like, ‘what I'm going to do is show a video to you and I’ll type in the thing that will keep the video on while I'm going to get the police. So, yeah, you have to think fast. (MHPi1)

Risk also related to safeguarding concerns. Remoteness rendered practitioners unable to ensure a confidential space, uncertain about the privacy of the space they were operating in and sometimes struggling to navigate obvious transgressions to confidentiality.

I've got one boy… I think [there are] some very dysfunctional adults who are piling into those conversations with us… I'm not entirely clear, because he doesn't put his camera on, whether he's got a conversation going alongside as well (MHPfg1).

[Patient’s] always been in her bedroom and at the end of the [third] video call, she shifted her laptop and there was a mirror on the wall behind her and there was somebody sat on the bed I could see reflected in the mirror… I don’t know how to address that… should I have said, ‘actually, this needs to be confidential and you need to be in a space we're not overheard’, or do I trust [patient’s] instincts and as long as they are happy in the environment (MHPi8)

#### Scope of care provision

Participants (particularly practitioners) discussed how delivering mental health services remotely may impact upon the scope of what can be offered, though it was evident that experiences varied according to the type of service provision that was being transferred for remote delivery and a range of types were represented within the sample from student counselling sessions, through family therapy and group sessions, to inpatient care.

Remoteness could increase accessibility because it offered a convenient and anonymous solution for those who are anxious or hard to reach. This extended to the inclusion of *‘dads in family therapy’* and working or separated parents who could *‘hop out for a video call’* (MHPfg2). Some found remoteness allowed them to extend their services. Improved continuity and an extended duration of care could be offered to students outside term-time as geographical constraints became unimportant; and meal support for eating disorders could be carried out at home and with parental involvement, instead of being limited to a clinical service setting. It was argued that remote appointments may be a tool for building independence and treating some young people at home when they might otherwise have been hospitalised.

There were one or two young people who were on the edge of needing specialist EDU [Eating Disorders Unit] so being really separated from their family at the time when they needed them most, but the Skype has enabled them to stay at home. I think that’s a huge benefit…for us to see what it was like in the family home, what the young person was experiencing, what the parents were having to cope with. (MHPfg2).

However, other practitioners argued that remoteness limited what they could deliver. They deemed some types of therapy highly problematic and were uncertain whether remote assessment could be carried out satisfactorily.

Certain things we can’t do, for example if you’re doing PTSD work, grounding is very difficult to do when someone’s not in the room. Exercises like mindfulness are very, very hard. Physically giving them objects to look at, focus on. (MHPi1).

Further, one practitioner implied that concerns about being able to contain risk (above) could limit the line of enquiry.

If I’m in a room with someone I can really gauge much better… ‘Do we need to kind of help them come back into a normal, you know, ‘this is the world we’re living in and what are you going to do today?’ If they’re already in this really chaotic situation, which many people are, I’m not going to push a question or something related to an issue that could be really distressing for them because I know that there’s nothing to contain it (MHPi4)

This led to a feeling of holding – ‘*literally just supporting’* (MHPfg2)—rather than progressing treatment for patients, which was also evident in the accounts of some young people and parents. Yet, for others, there was a sense that over time they had adapted to new, successful ways of working:

Actually what we’ve found is that we *are* treating people and we *are* bringing them forward in their recovery. Whilst we’re working differently, people are improving and they’re having positive experiences from their input. (MHPfg2)

## Discussion

This qualitative study explored how young people, parents and a range of practitioners experienced remote mental healthcare during the pandemic. Participants’ experiences reflected potential benefits and disadvantages. Primary concerns were about how remote appointments may hinder interpersonal communication, making therapeutic relationships more difficult to build or maintain and denying practitioners the opportunity to fully observe patients and draw on the nuances of non-verbal communication. Equally, participants were aware that remote communication compromised practitioner control, with implications for the way that young people engaged (or not) and practitioners’ abilities to ensure confidentiality, address safeguarding concerns and manage risk. This could create additional responsibilities for parents who gave examples of trying to mediate engagement and manage emotional fallout following difficult online appointments. For some young people, disadvantages were offset by the comfort of attending appointments from home and there was a sense that remote provision could make mental healthcare more accessible. However, others struggled with the boundaries blurred by bringing their mental health appointments into the home. Finally, the scope of remote appointments was debated, some believing this was limited to ‘holding’ but not progressing, while others cited examples of how it had allowed them to extend their services, provide naturalistic care and facilitate better involvement with parents. A narrative of learning and adaptation underlay many practitioner accounts.

Concerns around an impaired therapeutic relationship align with survey findings in which clinicians reported feeling less connected to their patients and that the flow of therapy is disrupted when not face-to-face [16, 19], and with broader concerns about how to build effective telemental health relationships with young people [10]. Participants in the current study went on to describe how this disconnection impeded the formation of trust and communication of empathy and reassurance. Despite this, some practitioners reported young people seeming more able to disclose during remote appointments due to the anonymity afforded. Disinhibition has been reported elsewhere in the literature, with service users feeling less fear of embarrassment or judgement in online therapy compared to face-to-face [14, 17, 31]_._ However, our data flag an important contradiction – that remoteness may facilitate disclosure while simultaneously hindering a practitioner’s capacity to respond to risk.

Young people endorsed the concern raised by practitioners in our study and elsewhere [22] about the difficulties of assessing the wellbeing of people living in challenging or unsafe environments or whose mental health is impacted by family/ home circumstances and who may feel uncomfortable discussing mental health from within the home. This underscores the potential limits to confidentiality when mental health appointments take place remotely in the absence of a safe, private space. The finding that other participants were able to benefit from the convenience, comfort and reduction in anxiety associated with attending appointments from home has also been reported elsewhere [17], though concerns about equitability arise for those without private space or the necessary technology to access online appointments [2, 13, 22]_._

Issues surrounding engagement with telehealth were reported pre-pandemic [32], but this study has provided fresh insights into the causes of disengagement and highlighted a range of disengaging behaviours due to the change in power dynamic when mental health appointments transition from being in the practitioners’ space to the private home of the young person. Young people were released from the formality of face-to-face appointments in service settings, while practitioners became virtual guests in young people’s homes. This could challenge expectations within the practitioner-patient relationship as it allowed young people to play an active role in determining how and whether they wished to engage. While one practitioner suggested a positive interpretation framed around patient autonomy, some young people and parents noted the disadvantages of being granted extra freedom to disengage and how poor engagement with online appointments could have repercussions for ongoing care. Such findings should be considered alongside suggestion that a switch to remote appointments may present a solution for non-attendance at in-person appointments [2]_._

Our findings also highlight that online service provision required parents/ carers to adopt stressful roles in relation to their child’s mental health care, and that they reported feeling unsupported in acquiring this greater responsibility. These included monitoring engagement in sessions, making adaptations to the physical environment to make it conducive to therapy, and assisting nervous children during appointments. Pre-pandemic research has endorsed the key role that parents typically play in managing their children’s mental health treatment attendance and adherence [33], which suggests this is not a new issue for parents, but that online appointments may compound existing pressures.

### Strengths and limitations

This study has provided a multi-perspective understanding by recruiting three key populations, covering those who deliver, have received, or who support young people to access remote mental healthcare. Previously, the experiences of young people and parents have not been well-documented. Use of an in-depth qualitative approach has added depth and further understanding to existing survey findings and responds to the need identified elsewhere for qualitative research in this area [2]. However, some sampling limitations were apparent since convenience sampling was used and participants were self-selecting. The number of parents recruited (*n* = 4) was limited, though each had detailed narratives to convey. Data on participants’ ethnicity were not collected. It is also possible that young people without access to private devices and stable connectivity were less likely to take part, which may have obscured considerations about equitability. The learning curve alluded to by some participants reflects the fact that data were collected during the first lockdown and indicates practice is likely to have evolved. Future research could usefully explore strategies employed to address challenges and enhance delivery of remote appointments in a post-pandemic context.

## Conclusion

Practitioners, parents and young people reported both positive and negative experiences associated with remote mental health support during the COVID-19 pandemic. Despite initial reluctance, it was apparent that practice was evolving, and most practitioners recognised contexts in which remote appointments could be useful. Promising features include the potential to make appointments more inclusive, accessible, naturalistic, and to extend the scope of practice. Some young people indicated similar views, referencing the benefits of being supported by home comforts during appointments. Many of the practitioners thought it likely their service would continue offering some remote appointments and early post-pandemic data indicate a possible long-term shift towards increasing remote appointments in place of face-to-face, while also noting that this is a largely unevaluated change in service provision [34]_._ Further research is needed to explore how remote services are best delivered, how practitioners can be supported, and the impact on young people’s outcomes. Our findings identify particular challenge areas to focus such research. Namely, how to mitigate against the reduced capability for practitioners to assess risk, greater burden on parents, tensions around mode of engagement and preservation of confidentiality, and constraints on interpersonal communication. Our findings further previous suggestion around the need to support practitioners with guidance on remote delivery and to develop skills to ensure successful implementation [5]_._

In the meantime, our findings suggest it is important for clinicians and service providers post-Covid, to consider whether and when it is appropriate to offer remote appointments as an alternative to, or alongside, face-to-face appointments. Personal choice of young people, families, and clinicians should be taken into account, alongside an awareness of circumstances in which remote appointments may be insufficient or even unsafe. It may be useful to begin working with families and young people in a face-to-face setting, before considering whether some or all of their appointments could be safely and effectively held remotely. Our work suggests that face-to-face appointments should be prioritised when engagement is more difficult, establishing and maintaining therapeutic relationships is more challenging, and especially when risks are identified around maintaining confidentiality in the home setting and safeguarding. There are also likely to be some therapeutic interventions which remain challenging to deliver remotely and will be more effectively delivered in face-to-face settings, and some patient circumstances that prevent access to this model of care.

We identified a clear narrative around the ways that clinicians learned and adapted to the sudden move to remote appointments. As clinicians and services become increasingly familiar with the practicalities of remote working, it will be important for discussion of and learning around the safe and effective use of remote sessions to continue.

## Data Availability

The datasets used and analysed during the current study are available from the corresponding author on reasonable request.
